# Change in Resting-State Functional Connectivity Following Working Memory Training in Individuals With Repetitive Negative Thinking

**DOI:** 10.1016/j.bpsc.2024.04.017

**Published:** 2024-05-04

**Authors:** Jessica Bomyea, Shirley Feng, Raeanne C. Moore, Alan N. Simmons, Michael L. Thomas

**Affiliations:** Center of Excellence for Stress and Mental Health, VA San Diego Healthcare System, San Diego, California (JB, ANS); Department of Psychiatry, University of California San Diego, San Diego, California (JB, SF, RCM, ANS); Massachusetts General Hospital, Athinoula A. Martinos Center for Biomedical Imaging, Boston, Massachusetts (SF); and the Department of Psychology, Colorado State University, Fort Collins, Colorado (MLT).

## Abstract

**BACKGROUND:**

Repetitive negative thinking (RNT) symptoms, which are characterized by pervasive, uncontrollable negative thoughts, are common in individuals with mood, anxiety, and traumatic stress disorders. Inability to regulate the contents of working memory is a hypothesized etiological factor in RNT, which suggests that training to improve working memory may be beneficial. This study examined the effects of working memory training on resting-state functional connectivity (rsFC) in individuals with elevated RNT and whether such changes would be associated with clinical improvement.

**METHODS:**

We conducted a secondary analysis of pre-post resting-state data collected as part of a randomized controlled trial (NCT04912089) of working memory training interventions (*n* = 42) compared with a waitlist control group (*n* = 23). We hypothesized that individuals who completed training would show increased rsFC between the 2 key intrinsic connectivity networks—the default mode network (posterior cingulate cortex) and the frontoparietal network (dorsolateral prefrontal cortex). We explored whether the magnitude of rsFC change was associated with change in RNT symptom severity.

**RESULTS:**

rsFC increased between the posterior cingulate cortex and regions including the frontal and parietal cortex in the training group compared with the waitlist group. Increased connectivity between the posterior cingulate cortex and superior frontal cortex was associated with RNT symptom reduction.

**CONCLUSIONS:**

These data provide evidence that working memory training can modulate neural circuitry at rest in individuals with RNT. Results are consistent with accounts of working memory training effects on large-scale neurocircuitry changes and suggest that these changes may contribute to clinical promise of this type of intervention on transdiagnostic RNT symptoms.

Repetitive negative thinking (RNT) is a common transdiagnostic symptom observed across stress-related disorders including mood, anxiety, and traumatic stress disorders (1). RNT subtypes manifest with varying content (e.g., rumination about past events, worry about future calamities) but share a common inability to regulate unwanted and affectively negative cognitions (2–4). Dysfunction of neurocognitive systems responsible for governing working memory contents is a key proposed mechanism of RNT, such that insufficient inhibition and removal of negative information from working memory manifests in being stuck in negative thinking (5–7). Working memory training (WMT) programs, designed to engage individuals in practiced, computer-administered exercises to enhance thinking skills, have shown promise for improving cognitive performance in depression (8), anxiety (9), and posttraumatic stress disorder (10,11). Relatively few studies have examined downstream clinical transfer of WMT specifically to RNT, and the results of those studies have been mixed; while links between WMT change and RNT have been shown in some studies, positive effects have not been universal (12–19). Thus, there remains a continued need to understand clinical impacts of WMT, including identifying mechanisms by which WMT programs may be helpful for RNT.

WMT effects are thought to occur in part via neuroplasticity, such that training-related improvements in co-activation and communications between neural circuits transfer to untrained tasks and broader functional outcomes that also depend on these circuits (20). Training-related neural outcomes can be measured via changes in functioning of components within intrinsic connectivity networks (ICNs)—sets of spatially distinct regions that co-activate during specific task or rest states (21). ICNs commonly implicated in psychopathology include the default mode network (DMN) and the frontoparietal network (FPN). The DMN, key nodes of which include the posterior cingulate cortex (PCC), medial prefrontal cortex, and precuneus, is considered to be a task-negative network, i.e., sets of brain regions that are typically activated during introspection and self-referential thought (22,23). In contrast, the FPN is considered to be one of multiple task-positive networks, i.e., sets of brain regions that typically increase in activation when an individual is engaged in activities that involve directing attention externally (24). The FPN, including nodes in the dorsolateral prefrontal, inferior frontal, and parietal cortices, is engaged by top-down cognitive control tasks that involve decision making and working memory (25). The salience network is also a task-positive network responsible for conflict detection and attentional orienting to salient cues, including processing of emotional cues, and includes the insula, anterior cingulate, and amygdala (26,27). Neuroimaging studies with nonpsychiatric samples have demonstrated that WMT modulates connectivity within task-positive networks (e.g., node of the FPN including the dorsolateral prefrontal cortex [dlPFC]), as well as between task-negative and task-positive networks (28,29).

Dysfunction in regulating the balance between internal and external cognitive processing, typically via DMN and FPN activity, may be relevant for understanding WMT-related neural effects on RNT. Greater RNT is associated with hyperconnectivity within the DMN in healthy individuals and individuals with depression (30–33). Moreover, abnormalities in cross-network resting-state functional connectivity (rsFC) are associated with RNT, including lower rsFC between the DMN and FPN circuitry (32,34–36), as well as greater rsFC between parts of the FPN and the salience network (37,38) and visual processing hubs [e.g., lingual gyrus (36)]. In one of few studies to date that have examined the impact of WMT on ICN connectivity in relation to RNT, Pan *et al.* found that brief WMT administered to individuals with elevated trait anxiety resulted in lower rumination and greater DMN connectivity as measured by electroencephalogram microstate recordings (39). The extent to which WMT results in similar changes as measured across ICNs in clinical samples characterized by elevated RNT remains to be established, but together these data suggest that such interventions can modulate the rsFC of networks that typically govern the balance of focus on introspection versus external thought.

The goal of the current study was to examine the effects of WMT on rsFC in individuals with mood, anxiety, and/or traumatic stress disorders with elevated RNT. To do this, we analyzed resting-state scan data collected before and after training as part of a randomized controlled trial of WMT versus a waitlist (WL) control group. Primary outcome data from this trial indicated that WMT beneficially impacted working memory performance and task-based functional neuroimaging outcomes (NCT04912089; J. Bomyea, Ph.D., *et al*., unpublished data, June 2024). We sought to extend this previous work by evaluating the extent to which there were changes in functional connectivity from key nodes of DMN and FPN circuits, based on previous work highlighting the role of the DMN and FPN in RNT and WMT, and whether such changes would be associated with clinical change. We hypothesized that individuals who completed WMT would show increased rsFC between the DMN and FPN regions and that the magnitude of change would be associated with change in RNT symptom severity.

## METHODS AND MATERIALS

### Participants

Participants were enrolled between October 2021 and May 2023. Inclusion criteria were 1) ages 21 to 55 years; 2) fluent in English; 3) met diagnostic criteria for one or more mood, anxiety, or traumatic stress disorders; 4) had a score above the clinical cutoff (321) on the Repetitive Negative Thinking Questionnaire-10 (40); 5) outpatient status; and 6) 6-week stability if taking a selective serotonin reuptake inhibitor. Exclusion criteria were 1) a diagnosis of severe alcohol use disorder during the past year, 2) a moderate or greater substance use disorder during the past year, 3) a lifetime history of psychotic or bipolar disorder, 4) acute suicidality necessitating immediate clinical intervention, 5) neurodegenerative or neurodevelopmental disorders, 6) a history of a moderate or severe traumatic brain injury or other known neurological condition, 7) sensory deficits that would preclude completing tasks, 8) conditions unsafe for completing magnetic resonance imaging (MRI) scanning (e.g., metal in the body, pregnancy), 9) currently receiving psychosocial treatment, and 10) currently receiving psychiatric pharmacotherapy, except selective serotonin reuptake inhibitors. Participants were recruited from community settings via posted flyers and digital advertisements. Inclusion criteria were evaluated using the Mini-International Neuropsychiatric Interview for DSM-5 (version 7.0.2) (41) and the PhenX Anxiety Disorders Screener (Composite International Diagnostic Interview Screening Scales) (42). All participants provided written informed consent, and the protocol was approved by the University of California San Diego Institutional Review Board and an independent data monitor prior to enrollment commencement. All procedures involving human participants were performed in accordance with the ethical standards of the University of California San Diego Human Research Protection Program and the Code of Ethics of the World Medical Association (Declaration of Helsinki).

A total of 68 individuals participated in a resting-state scan, 3 of whom were removed from the sample due to quality control metrics (1 with >15% of data censored, 2 with excess motion based on derivative or root mean square variance over voxels values), which resulted in a final sample of 65. See Table 1 for sociodemographic and clinical characteristics.

### Treatment

#### Working Memory Training.

The WMT is a modified computer-administered Rspan task (11,43) where participants were instructed to remember items presented while simultaneously solving a secondary processing task in which they decided whether a sentence was logically correct (11). First, a fixation cross was presented on the screen for 500 ms, followed by a sentence (e.g., “Jane walks her car in the park”). Once the participant indicated that they had read the sentence, they were shown a screen with 2 boxes for “True” or “False” to indicate whether the sentence was semantically logical or not. The following screen showed a letter for the participant to remember (500 ms). Sets of sentences and letters continued until the end of the trial, when participants were shown a recall screen with 12 letters and were instructed to select the letters that were previously shown in correct serial order. Participants completed 3 blocks of training during each session, with sessions lasting 30 to 45 minutes total. Within each block, participants trained on span sizes of 2 to 6, with 3 repetitions of each span size presented in random order such that during each of the 3 blocks, the participants completed 60 trials (3 sets of 2, 3 sets of 3, etc.). Participants were randomly assigned to complete either 2 sessions per week (8-session condition) or 4 sessions per week for 4 weeks (16-session condition).

#### Waitlist.

WL participants completed assessments at time points consistent with the pretraining, midtraining (week 2), and posttraining (week 5) assessments completed by the WMT group.

### Clinical Self-Report Measures

Baseline depression was measured with the Quick Inventory of Depressive Symptomatology (44), a 16-item self-report measure with each item rated on a 4-point Likert scale, with higher scores indicating greater depression severity. Baseline generalized anxiety severity was measured with the Generalized Anxiety Disorder-7 (45). It contains 7 items rated on a 4-point Likert scale, with higher scores suggesting more severe generalized anxiety symptoms. RNT outcomes were measured at pretraining, midtraining (2 weeks post baseline), and posttraining (4 weeks post baseline) using 2 self-report assessments given the transdiagnostic nature of the sample (1 each for worry and rumination). Rumination was measured with the Ruminative Response Scale (46). The Ruminative Response Scale is a 22-item self-report measure with each item rated on a 4-point Likert scale, with higher scores suggesting a higher level of rumination. Worry was assessed with the Penn State Worry Questionnaire (47), which comprises 16 items rated on a 5-point Likert scale and with higher scores suggesting greater worry severity. The total scales for each measure were *z*-standardized and averaged to create a composite score capturing RNT.

### Procedure

All participants provided informed written consent and completed clinical interviews and self-report measures prior to initiating training during an initial screening visit. Individuals who met inclusion criteria completed a baseline assessment visit that included functional MRI (fMRI). Following completion of this visit, participants were randomized 1:1:1 to WMT (8 or 16 sessions) or WL using a randomly permuted block design and stratified by primary diagnosis (anxiety/posttraumatic stress disorder or depression) and age (dichotomized <30 or ≥30 years). After the training phase, participants completed 2 posttraining assessment visits during which they completed clinical and fMRI assessments again. Participant compensation was the same in both groups and included up to $250 for completing all parts of the study. Results showing that WMT improved cognitive performance and modulated neural activity on task-based fMRI, as well as dose comparison, have been reported elsewhere (J. Bomyea, Ph.D., *et al*., unpublished data, June 2024); based on these earlier results, the 2 doses were collapsed for all analyses presented here.

### Analysis Plan

#### Single Participant Analysis.

Acquisition details can be found in Section S1 in the Supplement. The data were preprocessed and normalized to Montreal Neurological Institute coordinates using ANTsR, a statistical interface between Advanced Normalization Tools Software, R software, and AFNI. Steps included in fMRI preprocessing consisted of the removal of temporal outliers (AFNI: 3dDespike), field in-homogeneity correction (ANTsR: abpN4), slice time correction, and temporal whitening with bandpass filtering (ANTsR: preprocessing; 0.01 to 0.1). Motion correction and CompCor estimation correction were also included as part of this processing pathway, as well as spatial smoothing using Perona-Malik anisotropic diffusion (48), and motion and CompCor correction regressors were removed as part of the preprocessing steps prior to bandpass filtering. Outlying acquisitions (AFNI: 3dToutcount) and the first 10 repetition times were censored from the time series, as were acquisitions with large framewise displacement (< 0.3). Data were aligned to individual anatomical and Montreal Neurological Institute template (ANTsR: antsRegistration/antsApplyTransforms) for group comparisons.

#### Group-Level Analysis.

To investigate changes in the connectivity of nodes within the DMN and FPN, 1 seed was selected within each (PCC and dlPFC; 6-mm spheres). Region of interest (ROI) coordinates for the PCC-DMN seed (x = −4, y = −54, z = 1) were selected from a meta-analysis of functional neuroimaging studies of resting-state correlates of RNT (49), and coordinates for the dlPFC seed (x = −31, x = 46, z = 23) were selected from previous work that identified neural substrates of RNT within the FPN (32). Motion-corrected time series data were extracted for each ROI and used to create a functional connectivity map for each participant of *z*-transformed correlations between mean blood oxygen level–dependent time series of the ROI with all other voxels in the brain (AFNI: 3dDeconvolve). Group-level analyses comparing functional connectivity between individuals in the WMT and WL groups pre- to posttraining were conducted using AFNI’s 3dLME. AFNI’s updated 3dClustSim (50) was used to correct for multiple comparisons based on Monte Carlo simulation. 3dClustSim accounts for imaging volume and uses estimates of the spatial smoothness of the noise. Smoothness parameters were calculated using 3dFWHMx with the -acf option, which determines spatial autocorrelation as a function of radius and produces 3 parameters (a, b, c) that are averaged and input into 3dClustSim (0.715497, 1.56824, 4.86969). Analyses indicated that α < 0.05 was achieved using a voxel threshold of *p <* .005 with a minimum cluster size of 18 voxels.

#### Associations Between Change in rsFC and Change in RNT.

Parameter estimates from statistically significant clusters were extracted to visualize the directionality of results and to conduct correlation analyses. We sought to evaluate associations between pre-post change in clinical symptom outcomes with change in rsFC within statistically significant clusters separately within groups. To reduce the number of multiple comparisons, we limited our analyses to ROIs where significant zero-order Spearman correlations were observed in the full sample (uncorrected *p <* .05); this resulted in 1 ROI that was then further examined overall and separately within the WL and WMT groups using growth curve models to determine the relationships between rsFC and change in RNT symptoms over time. Models were conducted in SPSS version 23.0 (IBM Corp.) using restricted maximum likelihood estimation procedures and included effects of time (centered at posttraining), change in functional connectivity, and an interaction of time and change in functional connectivity, controlling for baseline generalized anxiety (Generalized Anxiety Disorder-7) and depression (Quick Inventory of Depressive Symptomatology) severity, with participant included as a random effect.

## RESULTS

### Sociodemographic and Clinical Features

There were no statistically significant differences between groups on baseline sociodemographic or clinical characteristics (Table 1). The sample was predominantly female, non-Hispanic White, and college educated, with depression and generalized anxiety disorder being the most common clinical disorders. Results showing that WMT outperformed WL on cognitive outcomes are reported separately and are summarized in Section S2 in the Supplement.

### Changes in rsFC Between WMT and WL Groups

**Figure 1.**
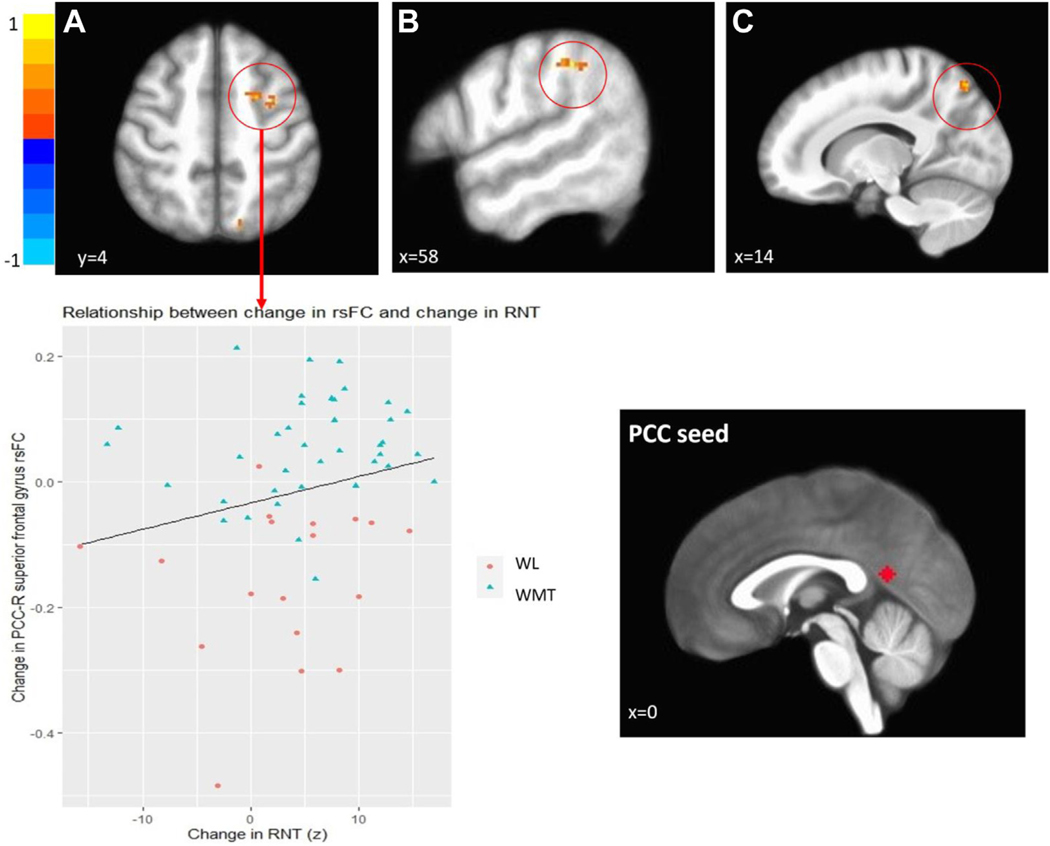
Regions demonstrating change in resting-state functional connectivity (rsFC) associated with the posterior cingulate cortex (PCC) seed from pre- to posttraining between the waitlist (WL) and working memory training (WMT) groups included the **(A)** right (R) superior frontal gyrus (region 1), **(B)** right superior parietal lobule (region 4), **(C)** right supramarginal gyrus (region 2) (other regions from Table 2 not shown; *p <* .005). Scatter plots showed that greater increase in functional connectivity between the PCC and right superior frontal gyrus was significantly correlated with greater improvement in repetitive negative thinking (RNT).

Areas of differential change in rsFC between the PCC seed and regions included the right superior frontal gyrus, right superior and inferior parietal lobule, right middle frontal gyrus, right precentral gyrus, and bilateral supramarginal gyrus. Functional connectivity increased in all regions in the WMT group, while decreases were observed in the WL group from pre- to posttraining (Table 2; Figure 1, top row). There was no evidence of statistically significant differences in dlPFC rsFC between the 2 groups from pre- to posttraining.

### Associations Between Change in rsFC and Change in RNT Symptoms

Change in connectivity between the PCC and right superior frontal gyrus was significantly correlated with change in RNT (*r* = 0.33, uncorrected *p <* .013), indicating that greater increase in functional connectivity was associated with greater reductions in RNT symptoms. No other associations reached statistical significance (*p*s >.15). We also examined WMT versus WL effects and correlations with rsFC separately for rumination and worry measures and found similar effects, with a slightly stronger response for rumination than worry (see Section S3 in the Supplement). Based on provisional evidence of an association between PCC–right superior frontal gyrus connectivity and RNT from the bivariate correlations, extracted data from this ROI was utilized in follow-up longitudinal models predicting RNT outcome improvements in RNT controlling for anxiety and depression. In the full sample, PCC–right superior frontal gyrus connectivity was associated with the trajectory (B = −0.88, SE = 0.35, *p* = .013) and posttraining RNT scores (B = −2.03, SE = 0.76, *p* = .009). Within the WMT group, results revealed that the effect of rsFC on trajectory did not reach statistical significance (B = −0.89, SE = 0.70, *p* = .21). However, an increase in rsFC connectivity was associated with lower posttraining RNT scores (B = −3.02, SE = 1.49, *p* = .0470). There was no association between rsFC and RNT in the WL group (RNT trajectory: B = −0.62, SE = 0.76, *p* = .42; posttraining RNT: B = −0.54, SE = 1.69, *p* = .75).

## DISCUSSION

In the current study, we investigated rsFC changes following administration of computerized WMT, a treatment that targets functions of working memory that are theorized to be cognitive mechanisms of RNT. Seed-based functional connectivity analysis was used with seeds in the DMN (PCC) and FPN (dlPFC) to examine potential changes in connectivity within or across ICNs relevant to RNT. In partial support of our hypotheses, results revealed differential changes in rsFC from the PCC, but not the dlPFC, between the WMT and WL groups from pre- to posttraining, with greater change in PCC–superior frontal gyrus connectivity being associated with symptom reduction.

The largest region that showed PCC-seeded rsFC changes was a lateral region of the superior frontal gyrus, with additional prefrontal cortical connectivity observed in the middle frontal and precentral gyri. These regions typically activate during working memory tasks (51,52) and belong to a frontoparietal subnetwork involved in regulatory control over processes like planning, maintaining task-relevant information, and following abstract rules (53). The PCC also demonstrated increased connectivity in the WMT compared with the WL group to inferior parietal regions critical for top-down attention, including anterior subregions implicated in sensorimotor processing and a middle subregion implicated in forming multimodal representations from somatosensory and visual cues (54,55). The PCC, in addition to being a core node of the DMN, is a functionally diverse region that coordinates cross-talk between ICNs and regulates attentional focus between internal and external thought (56–58). Taken together, rsFC from the PCC points to increased connectivity between the DMN and nodes of task-positive networks [i.e., frontoparietal and dorsal attention networks engaged by cognitively demanding or externally oriented tasks (24,59)] in individuals in the WMT group compared with those in the WL group. Observing rsFC changes seeded in this region, but not the selected dlPFC seed, suggests that it may be relatively more sensitive to training in the context of disorders characterized by RNT, which can be conceptualized as a disruption in the balance between internal and external thought.

The current study adds to a growing literature showing that WMT modulates neural functioning and extends this work by demonstrating broader connectivity effects at rest. Training-related changes have previously been observed in frontoparietal, cingulate, and visual processing region activity as well as task-based functional connectivity increases within frontoparietal and dorsal attention regions during active performance of working memory tasks (60). Because existing studies have predominantly examined neural consequences of training during cognitive tasks that are comparable to training paradigms, critiques have been raised about the broader applicability of acquired skills—namely, whether they exhibit effective transfer in both closely related (near-transfer) and more distantly related (far-transfer) tasks (61,62). The current approach using rsFC overcomes this limitation by capturing changes in intrinsic brain activity independent of a specific task or paradigm, thereby allowing for exploration of potential functional organization and network dynamic changes that may be altered by training. Group-based differences observed in rsFC, independent of task, are consistent with proposed network plasticity mechanisms of training transfer effects (63).

To our knowledge, the current study is the first to report WMT-related changes in rsFC in individuals with psychiatric disorders characterized by RNT. While task-positive and task-negative networks are typically anticorrelated at rest (24,64), exaggerated dysconnectivity may be problematic given that efficient interactions between them are critical for cognitive performance (65). Numerous studies have linked RNT to cross-network hypoconnectivity from the DMN (35,36), including a recent study that examined neural correlates of common transdiagnostic symptoms in individuals with mood, anxiety, and traumatic stress disorders that found that RNT was associated with problematic hypoconnectivity between the DMN and task-positive networks (66). This suggests that reintegrating connectivity across these networks may have beneficial restorative effects. The observation that greater RNT symptom change was associated with increased rsFC between the PCC and superior frontal gyrus is consistent with this proposal. We hypothesize that cross-network connectivity is repeatedly engaged during WMT across the DMN and other task-positive networks, the persistent effects of which include greater efficiency of large-scale functional activity in the brain that underlies effective regulation of introspective thought. By this account, training-related improvements in DMN–task-positive network coordination could improve the regulation of internal versus external attention that appears aberrant in RNT, potentially resulting in improved ability to disengage from negative thinking.

This study has several limitations. First, this study has a modest sample size, and the participants had relatively mild clinical symptoms; thus, our findings should be replicated with a larger sample of individuals with more severe symptoms. Evaluation of effects with a longer duration of follow-up will be needed to determine whether changes last over time. Because this was the first study of its kind to test this WMT intervention in the studied population and was not designed as an efficacy trial, we opted to use a WL comparison group to control for basic elements like the passage of time and repeated assessment (67). However, differences using a WL design could be due to negative effects related to waiting for an expected cognitive training program (e.g., disappointment about randomized condition that biased reporting of symptoms for those in the WL group, negative beliefs about WL assignment could impact neural activity) (68). There may also be between-group differences in nonspecific elements of computerized training (e.g., interfacing with the computer program) because some previous trials have found some beneficial effects of sham trainings (19,69). Thus, while results from the current study can provide provisional evidence of effects, additional studies with active control groups are needed. A seed-based analysis was utilized in our study to specifically investigate the DMN and FPN networks, and the potential involvement of other networks associated with WMT-leaded RNT changes might not have been detected. Future work examining alternative methods for exploring cross-ICN communication (e.g., graph theory metrics) and a more nuanced timescale of connectivity (e.g., dynamic functional connectivity) should be included to provide a more fine-grained analysis of changes. In addition, future work that does not limit exploration of relationships between change in RNT and change in ROIs to those that change differentially between groups should be conducted, as should larger scale studies that can elucidate brain-behavioral relationships, including the question of whether the relationship between rsFC and change in symptoms is stronger in individuals who receive training. Previous experience with cognitive training or rehabilitation was not considered exclusionary in the current study. While no participants spontaneously reported use of cognitive training during the review of treatment history, future studies should explicitly query participants about their experience with these programs to determine any effects of prior exposure.

### Conclusions

Despite these limitations, the current study provides initial evidence that WMT modulates functioning in neural circuitry relevant to RNT. The results add to a growing literature regarding the effects of WMT on neural outcomes and highlight the potential impact of WMT on neural and clinical markers in individuals with stress-related disorders and elevated RNT.

## Supplementary Material

SM

Supplementary material cited in this article is available online at https://doi.org/10.1016/j.bpsc.2024.04.017.

## Figures and Tables

**Table 1.** Sociodemographic and Clinical Characteristics

	WL, *n* = 23	WMT, *n* = 42	Statistical Comparison Between Groups at Baseline
Age, Years	31.85 (7.86)	32.67 (8.58)	*F*_1,64_ = 0.15, *p* = .70
Highest Education^a^			
Less than high school	0 (0%)	0 (0%)	Χ^2^5 = 5.38, *p* = .37
High school/GED	0 (0%)	2 (5%)	
Associates or equivalent degree	7 (3%)	8 (19%)	
College degree	11 (48%)	19 (46%)	
Graduate or professional study	5 (22%)	12 (29%)	
Sex			
Female	20 (87%)	32 (76%)	Χ^2^1 = 1.08, *p = .*30
Male	3 (13%)	10 (24%)	
Race			
Asian	2 (8.7%)	5 (12%)	Χ^2^4 = 0.91, *p = .*92
Black	0 (0%)	1 (2%)	
More than one race	5 (22%)	7 (17%)	
White	14 (61%)	25 (60%)	
Declined to answer	2 (9%)	4 (10%)	
Ethnicity			
Hispanic or Latino	7 (30%)	15 (36%)	Χ^2^1 = 0.19, *p = .*67
Not Hispanic or Latino	16 (70%)	27 (64%)	
Mean Baseline Depression Severity, QIDS	11.75 (4.49)	11.89 (4.33)	*F*_1,56_ = 0.01, *p* = .91
Mean Baseline Anxiety Severity, GAD-7	8.45 (4.65)	10.18 (5.36)	*F*_1,56_ = 1.50, *p* = .23
Baseline Diagnoses			
Panic disorder	3 (13%)	2 (5%)	–
Agoraphobia	1 (4%)	0 (0%)	
Social anxiety disorder	3 (13%)	9 (21%)	
Posttraumatic stress disorder	4 (17%)	8 (19%)	
Generalized anxiety disorder	9 (39%)	14 (33%)	
Depressive disorder	5 (22%)	11 (26%)	
Mean Worry Severity, PSWQ			
Pretraining	50.50 (5.93)	50.13 (6.67)	*F*_1,56_ = 0.04, *p* = .84
Midtraining	50.50 (7.30)	48.18 (7.01)	
Posttraining	48.98 (7.14)	47.11 (6.50)	
Mean Rumination Severity, RRS			
Pretraining	51.70 (12.39)	53.34 (12.99)	*F*_1,56_ = 0.22, *p* = .64
Midtraining	53.10 (11.82)	48.53 (12.24)	
Posttraining	50.70 (14.20)	46.55 (12.68)	

Values are presented as mean (SD) or *n* (%).

GAD-7, Generalized Anxiety Disorder 7-item scale; GED, General Educational Development credential; PSWQ, Penn State Worry Questionnaire; QIDS, Quick Inventory of Depressive Symptomatology; RRS, Ruminative Response Scale; WL, waitlist; WMT, working memory training.

a Data were missing for 1 participant.

**Table 2.** Areas of Differential Change in Resting-State Functional Connectivity (Posterior Cingulate Cortex Seed) Between WMT and WL Groups From Pre- to Posttraining

		MNI							
ROI	Voxels	x	y	z	*t* Statistic	Region	BA	WMT Mean Change	WL Mean Change

1	33	21	4	52	11.56	Right superior frontal gyrus	6	0.05 (0.08)	−0.16 (0.12)

2	29	13	−74	55	12.31	Right superior parietal lobule	7	0.06 (0.14)	−0.19 (0.16)

3	29	34	2	55	12.31	Right middle frontal gyrus	6	0.08 (0.14)	−0.17 (0.14)

4	26	59	−36	42	12.30	Right supramarginal gyrus (middle IPC)	40	0.10 (0.14)	−0.14 (0.10)

5	24	48	1	45	13.56	Right precentral gyrus/caudle middle frontal gyrus	6	0.08 (0.13)	−0.15 (0.11)

6	21	53	−24	34	13.44	Right supramarginal gyrus (anterior IPC)	2	0.05 (0.12)	−0.17 (0.13)

7	21	−60	−24	37	12.76	Left supramarginal gyrus (anterior IPC)	2	0.07 (0.12)	−0.17 (0.19)

Data are presented as mean (SD).

BA, Brodmann area; IPC, inferior parietal cortex; MNI, Montreal Neurological Institute. ROI, region of interest; WL, waitlist; WMT, working memory training.
