# Supplementary material for: Change in Resting-State Functional Connectivity Following Working Memory Training in Individuals With Repetitive Negative Thinking
Source: Biol Psychiatry Cogn Neurosci Neuroimaging. Author manuscript; Available in PMC 2025 Sep 16. (PMC12440368; doi:10.1016/j.bpsc.2024.04.017)
Supplement: SM [file NIHMS2080968-supplement-SM.pdf]

SUPPLEMENTARY INFORMATION

Change in Resting State Functional Connectivity Following Working Memory Training in Individuals With Repetitive Negative Thinking

Bomyea *et al.*

S1. MRI acquisition parameters

Participants were scanned in a 3T Siemens Prisma scanner using a 32-channel head array coil. Participants were instructed to relax and keep their heads still with their eyes open and to avoid falling asleep. Each scanning session included a three-plane scout scan, a sagittally acquired sequence for acquiring T1-weighted images (MPRAGE) [T1:FOV 240x256mm; matrix: 300x320; slices 208; slice thickness: 0.80mm; TR: 2400ms, TE: 2.22ms, flip angle: 8, inversion time: 1000ms] and one T2*-weighted axially acquired echo-planar imaging (EPI) scans to measure blood oxygen level dependent (BOLD) signals during rest [T2: 2.0x2.0x2.0mm voxel size; 104x104 acquisition matrix, TR: 800ms, TE: 37ms, flip angle: 52, multiband factor of 8, 72 acquired slices (whole brain)].

S2. Effect of WMT versus WL on neurocognitive outcomes.

To verify that training impacted working memory, groups were compared on an independent measure of working memory (Operation span; Ospan), behavioral data from a working memory task completed in-scanner (Reading span; Rspan) and a composite measure of fluid cognition (NIH Toolbox). Results from the analyses found that individuals in WMT showed greater improvements over time relative to WL on the Ospan $F(1,61)=4.42$, $p=.04$, $\eta_p^2=.07$, the Rspan, $F(1,58)=5.55$, $p=.02$, $\eta_p^2=.09$, and NIH Toolbox measure $F(1,57)=5.03$, $p=.03$, $\eta_p^2=.08$ (also reported in Bomyea et al., 2024).

S3. Comparing effects on rumination and worry outcomes separately

We examined whether WMT versus WL effects or associations with rsFC differed by construct (RNT composite versus the worry versus rumination components). Estimates of within group effects from a composite of worry and rumination measures of RNT demonstrated trend-level contrast-based reductions in the WMT group $F(1,41)=3.77$, $p=.059$, $\eta_p^2=.08$, that were not

observed in the WL group, $F(1,21)=.92$, $p=.349$, $\eta_p^2=.04$. Similarly for rumination, we found statistically significant reductions in the WMT group $F(1,41)=4.42$, $p=.042$, $\eta_p^2=.10$, that were not observed in the WL group, $F(1,21)=.93$, $p=.346$, $\eta_p^2=.04$. For worry, though the pattern of means was similar there was not a statistically significant effect in the WMT group, $F(1,41)=1.05$, $p=.31$, $\eta_p^2=.03$, or the WL group, $F(1,21)=.63$, $p=.44$, $\eta_p^2=.03$. In terms of associations with rsFC, changes in our RNT composite score were associated with changes in the right superior frontal gyrus, $r=.314$, $p=.013$. The magnitude of the relationship between changes in rsFC and changes in the RRS ($r=.307$, $p=.021$) and PSWQ ($r=.285$, $p=.033$) were similar in magnitude, though stronger for rumination than worry. Together, these data suggest that training effects and neural changes may be more associated with the rumination component of RNT versus the worry component.
